# Selective Dopamine Transporter Inhibitor CE-123 Modulates Region-Specific Intestinal Mucosal and Immune Responses Following Fear Conditioning and Extinction in Adolescent Female Rats

**DOI:** 10.3390/biomedicines14102199

**Published:** 2026-09-28

**Authors:** Katarzyna Kras, Małgorzata Manastyrska-Stolarczyk, Kamil Arciszewski, Cezary Osiak-Wicha, Paweł Grochecki, Jolanta H. Kotlińska, Tymoteusz Słowik, Gert Lubec, Marcin B. Arciszewski

**Affiliations:** 1 Department of Animal Anatomy and Histology, Faculty of Veterinary Medicine, University of Life Sciences in Lublin, Akademicka 12, 20-950 Lublin, Poland; katarzyna.kras@up.edu.pl (K.K.);; 2 Clinical Neurology Ward, The University Clinical Hospital No. 4 in Lublin, Jaczewskiego 8, 20-090 Lublin, Poland; 3 Department of Pharmacology and Pharmacodynamics, Medical University of Lublin, Chodzki 4a, 20-093 Lublin, Poland; 4 Experimental Medicine Center, Medical University of Lublin, Jaczewskiego 8, 20-090 Lublin, Poland; 5 Department of Neuroproteomics, Paracelsus Medical University, 5020 Salzburg, Austria

**Keywords:** fear conditioning, fear extinction, intestinal mucosa, MUC2, mucosal immune response, dopamine transporter, CE-123, post-traumatic stress disorder

## Abstract

**Background/Objectives:** Post-traumatic stress disorder (PTSD) is associated with systemic alterations that may involve the gastrointestinal tract, yet intestinal responses to fear-related learning remain poorly characterized. We investigated intestinal mucosal morphology, MUC2 immunoreactivity, and local immune responses following fear conditioning and extinction, and assessed their modulation by the selective dopamine transporter inhibitor CE-123. **Methods:** Twenty-four adolescent female Wistar rats were assigned to Control or fear-conditioned (FC) groups receiving Vehicle or CE-123 (10 mg/kg, i.p.) during the extinction phase. Morphometry and immunohistochemical detection of CD68, CD206, iNOS, IL-6, IL-10, and MUC2 were evaluated in the duodenum, jejunum, ileum, and colon. **Results:** Fear conditioning was associated with marked region-dependent alterations. Among Vehicle-treated animals, villus height was increased in the duodenum and jejunum, accompanied by enhanced immunoreactivity of several inflammation-associated markers. In contrast, the ileum and colon showed distinct profiles characterized mainly by increased IL-6, reduced selected immunoregulatory markers, and decreased MUC2. CE-123 in fear conditioning (FC) showed its clearest attenuation of inflammation-associated responses in the jejunum, where CD68, iNOS, and IL-6 immunoreactivity were reduced. In comparison, this pattern was not consistently observed in other intestinal regions: in the duodenum, only CD68 immunoreactivity was reduced, whereas in the ileum, a reduction was observed only for IL-6. In the colon, both IL-6 and CD68 showed changes in the opposite direction to those observed in the jejunum. Collectively, these region-specific and divergent responses suggest that CE-123 exerts a modulatory rather than uniformly anti-inflammatory effect on intestinal immune responses. CE-123 also produced independent effects in Control animals, including segment-specific changes in mucosal morphometry, MUC2, and selected immune markers, indicating that its actions were not limited to attenuation of conditioning-associated alterations. **Conclusions:** These findings demonstrate segment-specific intestinal remodeling following fear conditioning and extinction and indicate a context-dependent immunomodulatory effect of CE-123.

## 1. Introduction

Post-traumatic stress disorder (PTSD) is a complex trauma-related disorder that may develop following exposure to a traumatic event [1,2]. Although PTSD is primarily defined by persistent psychological symptoms, a growing body of evidence indicates that it should also be considered in the context of its systemic consequences. Individuals with PTSD show increased susceptibility to immune, gastrointestinal, cardiovascular, and musculoskeletal disorders, as well as a higher risk of comorbid psychiatric conditions, including depression, anxiety disorders, and substance use disorders [2,3]. Exposure to potentially traumatic events is common in the general population, whereas PTSD develops only in a subset of trauma-exposed individuals, suggesting that individual vulnerability and regulation of the stress response play key roles in its pathophysiology [4,5].

The biological response to traumatic stress is regulated primarily by neuroendocrine and autonomic mechanisms under hypothalamic control. These include the rapidly acting sympathetic-adrenal-medullary system, which is responsible for the immediate mobilization of the organism, and the hypothalamic–pituitary–adrenal (HPA) axis, which stimulates glucocorticoid release and contributes to the long-term regulation of metabolic and immune processes [3,6]. In PTSD, persistent dysregulation of these stress-response systems may contribute to chronic immune activation and impaired function of peripheral organs. The gastrointestinal tract is particularly relevant in this context because it is highly responsive to neuroendocrine, autonomic, and immune signals and may therefore represent an important peripheral target of PTSD-related disturbances [7,8,9].

Experimental studies of trauma-related disorders commonly use fear-conditioning and extinction procedures to investigate selected mechanisms relevant to the acquisition, persistence, and regulation of aversive memories. Such paradigms do not reproduce the full clinical complexity of PTSD but provide a controlled approach for studying neurobiological processes implicated in trauma-related responses [10]. Although the neural mechanisms of fear conditioning and extinction have been extensively investigated, considerably less is known about their peripheral consequences, including their effects on the gastrointestinal tract. Most available data on stress-related intestinal alterations derive from models of chronic or generalized psychological stress. These studies indicate that stress can induce structural alterations in the intestinal mucosa, including changes in villus height, crypt morphology, and epithelial renewal [11,12], as well as modifications of the local immune microenvironment and inflammatory responses [13]. Intestinal alterations have also been reported following severe or repeated psychosocial stress, including disruption of mucosal barrier homeostasis and enhanced local inflammatory responses [14]. However, intestinal mucosal responses following fear conditioning and extinction, particularly their regional variation along the gastrointestinal tract, remain poorly characterized.

The intestinal mucosa is a dynamic environment in which the epithelium, mucus layer, and local immune cells and mediators act together to maintain tissue homeostasis [15,16]. MUC2, the major gel-forming mucin and mucosal component of the intestinal barrier produced by goblet cells, is a key component of intestinal mucus and plays an important role in maintaining mucosal homeostasis [15,17]. Importantly, *Muc2* expression is sensitive to stress-related alterations; repeated exposure to water-immersion stress in rats reduced colonic *Muc2* expression and, after prolonged exposure, was also associated with a decrease in goblet cell numbers [18]. At the same time, cytokines such as IL-6 and IL-10, together with markers associated with the monocyte–macrophage compartment, including CD68, iNOS and CD206, can provide complementary information on the local immune profile of the intestinal mucosa [19,20,21]. Therefore, the combined assessment of mucosal morphometry, MUC2 immunoreactivity, and selected immune markers may provide a more comprehensive picture of intestinal responses following fear conditioning and extinction.

Dopaminergic signaling is involved in the regulation of both fear extinction and gastrointestinal function [22,23]. Modulation of dopamine availability during extinction may therefore influence not only central mechanisms of fear regulation but also peripheral responses mediated through brain–gut interactions. In addition, functional dopamine transporter (DAT) expression has been reported in monocytes and macrophages, including a subset of intestinal macrophages [24] and dopamine can modulate cytokine production in immune cells depending on receptor subtype, dopamine concentration, and cellular activation state [25]. These observations provide a potential link between DAT inhibition and modulation of the intestinal immune environment. CE-123 is a selective inhibitor of the DAT, which is responsible for the reuptake of dopamine from the extracellular space [26]. Because CE-123 was administered during the fear-extinction phase of the present paradigm, its effects provide an opportunity to examine whether selective DAT inhibition is associated with changes in intestinal responses following prior aversive conditioning. Therefore, the aim of the present study was to assess intestinal mucosal morphometry, MUC2 immunoreactivity, and local immune responses in the duodenum, jejunum, ileum, and colon following fear conditioning and extinction in adolescent female Wistar rats, and to determine whether these responses are modulated by selective dopamine transporter inhibition with CE-123.

We hypothesized that prior aversive fear conditioning followed by extinction would be associated with segment-specific alterations in intestinal mucosal morphology and local immune responses, including changes in markers related to inflammatory and immunoregulatory processes and in MUC2 immunoreactivity. We further hypothesized that selective dopamine transporter inhibition with CE-123 during fear extinction would modulate these intestinal responses in a segment-dependent manner.

## 2. Materials and Methods

### 2.1. Animals and Experimental Design

Twenty-four adolescent female Wistar rats (OMD, Lublin, Poland) were used in the study. At the beginning of the experiment, the animals were 28 days old. Behavioral procedures were initiated on postnatal day 30, at which time body weight ranged from 112 to 150 g. Animals were randomly allocated to four experimental groups (*n* = 6 per group): Control + Vehicle, Control + CE-123, FC (fear conditioning) + Vehicle, and FC + CE-123. No formal *a priori* sample-size calculation was performed. The group size was established before the experiment based on previous experience with comparable in vivo paradigms and with consideration of the reduction principle of the 3Rs.

The animals were housed in individually ventilated cages, with three rats per cage, and had *ad libitum* access to standard laboratory chow and water. Environmental enrichment included nesting material, tunnels, and wooden blocks. Animals were maintained under controlled environmental conditions, with a temperature of 20–24 °C, relative humidity of 55 ± 10%, and a 12 h light/12 h dark cycle. The noise level in the animal housing room did not exceed 60 dB. Behavioral procedures were conducted between 08:00 and 19:00.

Animals were monitored daily throughout the experiment for general health and welfare and for signs of pain or distress. Humane endpoints were defined in accordance with the protocol approved by the Local Ethics Committee for Animal Experiments. No adverse events were observed, and all animals completed the experimental protocol.

All experimental procedures were approved by the Local Ethics Committee for Animal Experiments (approval no. 11/2024, 26 February 2024) and were conducted in accordance with Directive 2010/63/EU, issued on 22 September 2010. The study adhered to the ARRIVE 2.0 guidelines for reporting in vivo experiments.

### 2.2. Fear Conditioning and Extinction Paradigm

An experimental behavioral paradigm incorporating fear conditioning, sensitization, extinction, and reinstatement was used. Following a two-day handling period, animals underwent fear conditioning on experimental day 1 (postnatal day 30) in a sound-attenuated conditioning chamber (Ugo Basile S.R.L., Gemonio, Italy). In context A, animals assigned to the fear-conditioned (FC) groups were exposed twice to a 30 s auditory cue (80 dB), with a 2 s foot shock (1.5 mA) delivered during the final 2 s of each cue. Control animals were exposed to the same context and auditory cues without the 1.5 mA foot shock. The terms Control and FC distinguish animals according to the presence or absence of associative fear conditioning. In the FC groups, the auditory conditioned stimulus (CS) was paired with foot shock as the unconditioned stimulus (US), establishing a CS–US association. Control animals were exposed to the auditory stimulus but did not receive CS–US pairing. Thus, although Control animals participated in the subsequent experimental procedures, they had not acquired the conditioned association between the auditory cue and foot shock.

Following an 11-day interval without behavioral procedures, all animals were exposed on experimental day 13 to a distinct context B and received three 1 s foot shocks of lower intensity (0.4 mA) to assess sensitization to an aversive stimulus. On day 14, conditioned responses to contexts A and B were assessed in the absence of foot shock, and anxiety-like behavior was evaluated using the elevated plus maze on day 15. Fear extinction was performed on days 17–20 by repeated exposure to context A and the auditory cue without foot shock. On day 21, all animals were re-exposed to context A and received a low-intensity foot shock (0.15 mA), followed by a final cue-exposure session without foot shock on day 22. Behavioral procedures were performed without blinding to experimental group allocation because the conditioning protocol differed between groups.

Behavioral outcomes were recorded as part of the broader experimental project but were designated for a separate analysis and are not reported in the present study, which was focused on intestinal morphometric and immunohistochemical outcomes. Accordingly, the Control and FC designations used here refer to the experimental conditions to which the animals were assigned rather than to classification based on individual behavioral response criteria.

### 2.3. CE-123 Administration

The S-enantiomer of CE-123 [5-((benzhydrylsulfinyl)methyl)thiazole], a thiazole-based modafinil derivative and selective dopamine transporter inhibitor, was provided by the Lubec Laboratory (University of Vienna, Vienna, Austria). The compound was synthesized according to previously published procedures and showed high chemical and enantiomeric purity (99.7%), as determined by HPLC analysis [27]. From experimental day 17 to day 22, animals assigned to the CE-123-treated groups received S-CE-123 intraperitoneally (i.p.) once daily at a dose of 10 mg/kg body weight, 30 min before the respective behavioral procedure. The compound was freshly prepared on each day of administration in a vehicle containing 1% DMSO and 3.3% Tween 80 in 0.9% NaCl. Animals in the Control + Vehicle and FC + Vehicle groups received a corresponding volume of the drug-free vehicle according to the same administration schedule. Treatment administration was not performed under blinded conditions.

### 2.4. Sample Collection and Tissue Processing

Immediately after completion of the final behavioral session on experimental day 22, the animals were euthanized by decapitation. Tissue samples were collected from the duodenum, jejunum, ileum, and colon, gently rinsed with physiological saline to remove residual luminal contents, and immediately fixed in 10% neutral buffered formalin for 24 h at room temperature. Following fixation, the samples were rinsed in running tap water, dehydrated through a graded ethanol series, cleared in xylene, and infiltrated with Paraplast. The tissues were subsequently embedded in paraffin blocks using a modular tissue embedding system (MYR EC-350, Casa Álvarez Material Científico SA, Madrid, Spain). Paraffin-embedded intestinal samples were sectioned on a rotary microtome (HM 360, Microm, Walldorf, Germany) into 3-µm-thick sections and mounted on microscope slides for subsequent histological and immunohistochemical analyses.

### 2.5. Histological Staining and Morphometric Analysis

For histological assessment, paraffin-embedded intestinal sections were deparaffinized in xylene, rehydrated through a descending ethanol series, and stained using the Masson–Goldner trichrome method (Masson-Goldner staining kit 1004850001, Merck, Darmstadt, Germany). Following staining, the sections were dehydrated through an ascending ethanol series, cleared in xylene, and mounted for microscopic evaluation.

Microscopic evaluation was performed using a BX-51 light microscope (Olympus, Tokyo, Japan) equipped with a DP-70 digital camera (Olympus, Tokyo, Japan). Digital images were acquired using Cell^M 2.3 software (Olympus, Tokyo, Japan) and subjected to morphometric analysis using ImageJ (v1.54g) software (National Institutes of Health, Bethesda, MD, USA). In the duodenum, jejunum, and ileum, villus height and width and crypt depth and width were measured. Villus height was determined from the tip of the villus to the villus–crypt junction, whereas villus width was measured at the midpoint of the villus. Crypt depth was measured from the crypt opening to its base, and crypt width was determined at the midpoint of the crypt. In the colon, crypt depth and width were assessed using the same criteria. Only intact, well-oriented villi and crypts showing a longitudinal profile and preserved mucosal architecture were included in the analysis. For each animal, morphometric measurements were obtained from one representative histological section from each intestinal segment. In the duodenum, jejunum, and ileum, 10 intact, well-oriented villi and 10 corresponding crypts were measured per section, whereas 10 well-oriented crypts were evaluated in the colon. Measurements obtained from individual structures were averaged to generate a single value for each morphometric parameter per animal, and the animal was considered the experimental unit. Morphometric measurements were performed by an investigator blinded to experimental group allocation.

### 2.6. Immunohistochemical Analysis

Immunohistochemical staining was performed on paraffin-embedded intestinal sections using the DAB (3,3′-diaminobenzidine) chromogen method. Sections were deparaffinized in xylene and rehydrated through a descending ethanol series to distilled water. After washing in PBS, heat-induced epitope retrieval was performed in citrate buffer (pH 6.0) using a pressure cooker (8 min, 120 °C; multicooker RMC-PM381-E, Redmond Industrial Group, Guangzhou, China). The slides were then allowed to cool, and endogenous peroxidase activity was blocked by incubation with 3% hydrogen peroxide for 15 min. After washing in PBS containing Tween-20, the sections were incubated with PBS containing Triton X-100 for 10 min and subsequently washed again in PBS with Tween-20.

The sections were incubated overnight at 4 °C with primary antibodies against CD68, CD206, inducible nitric oxide synthase (iNOS), interleukin-6 (IL-6), interleukin-10 (IL-10), and mucin 2 (MUC2) (Table 1). On the following day, the sections were washed in PBS with Tween-20 and incubated with a two-step detection system (ImmunoLogic, Arnhem, Netherlands). A post-antibody blocking reagent was applied for 15 min, followed by washing in PBS with Tween-20. The sections were then incubated for 30 min with a horseradish peroxidase (HRP)-conjugated secondary detection reagent.

After subsequent washing, the immunoreaction was visualized using a DAB substrate kit (ab64238; Abcam, Cambridge, UK) for 1 min. The sections were counterstained with haematoxylin (Patho, Mar-Four, Konstantynów Łódzki, Poland), rinsed in running tap water, dehydrated through an ascending ethanol series, cleared in xylene, and mounted for microscopic evaluation.

Primary antibodies were selected based on manufacturer-provided information supporting their use in immunohistochemical applications and documented species reactivity and/or previous use in rat samples. These data supported application- and species-level antibody selection but did not constitute independent validation of each antibody specifically in rat intestinal tissue. For each immunohistochemical reaction, negative-control sections were processed in parallel by omitting the primary antibody and replacing it with PBS [28,29].

### 2.7. Quantitative Image Analysis

Digital images obtained after immunohistochemical staining were analyzed using ImageJ software (version 1.54g, National Institutes of Health, Bethesda, MD, USA). Images were acquired under identical microscope, camera, magnification, illumination, and exposure settings for all experimental groups within a given immunohistochemical reaction. Image analysis was performed by an investigator blinded to the experimental group allocation.

For each image, the intestinal mucosa was manually delineated as the region of interest (ROI). Colour deconvolution was performed using the H DAB vector to separate the haematoxylin and DAB components, and the DAB channel (Colour_2) was used for quantitative analysis. DAB-positive staining was quantified using threshold-based segmentation. Threshold settings were optimized separately for each immunohistochemical marker to distinguish specific DAB staining from background, and the same threshold value established for a given marker was subsequently applied to all images analyzed for that marker.

For each marker and each intestinal segment, 10 non-overlapping microscopic fields per animal were analyzed. The percentage of immunoreactive area within each ROI was calculated as the area fraction of pixels classified as DAB-positive relative to the total ROI area and expressed as %Area. Quantitative analyses were performed separately for the duodenum, jejunum, ileum, and colon. Measurements from the 10 microscopic fields were averaged within each animal to obtain a single representative value for each marker and intestinal segment. The individual animal was used as the experimental unit for statistical analysis.

### 2.8. Statistical Analysis

Statistical analyses were carried out in GraphPad Prism version 10.4.1 (GraphPad Software, Boston, MA, USA). Results are expressed as mean ± SEM, with each animal contributing a single value to the statistical analysis (*n* = 6 per group). For morphometric and immunohistochemical measurements, values obtained from multiple structures or microscopic fields within the same animal were averaged before analysis.

The duodenum, jejunum, ileum, and colon were analyzed independently. Within each intestinal segment, each morphometric and immunohistochemical outcome was analyzed using a two-way ANOVA with fear conditioning (Control vs. FC) and treatment (Vehicle vs. CE-123) as fixed factors, including the interaction between these factors. Residual distributions and residual-versus-fitted plots were inspected to assess the assumptions and suitability of the ANOVA model.

For each outcome within each intestinal segment, four prespecified pairwise comparisons were performed to assess the effect of CE-123 separately in Control and FC animals and the effect of fear conditioning separately in Vehicle- and CE-123-treated animals. These four prespecified comparisons were considered a single family of comparisons, and their *p*-values were adjusted using the Holm-Bonferroni procedure. No additional multiplicity correction was applied across different intestinal segments or across distinct morphometric and immunohistochemical outcomes. Statistical significance was set at *p* < 0.05.

## 3. Results

### 3.1. Intestinal Mucosa Morphometry

**Figure 1:**
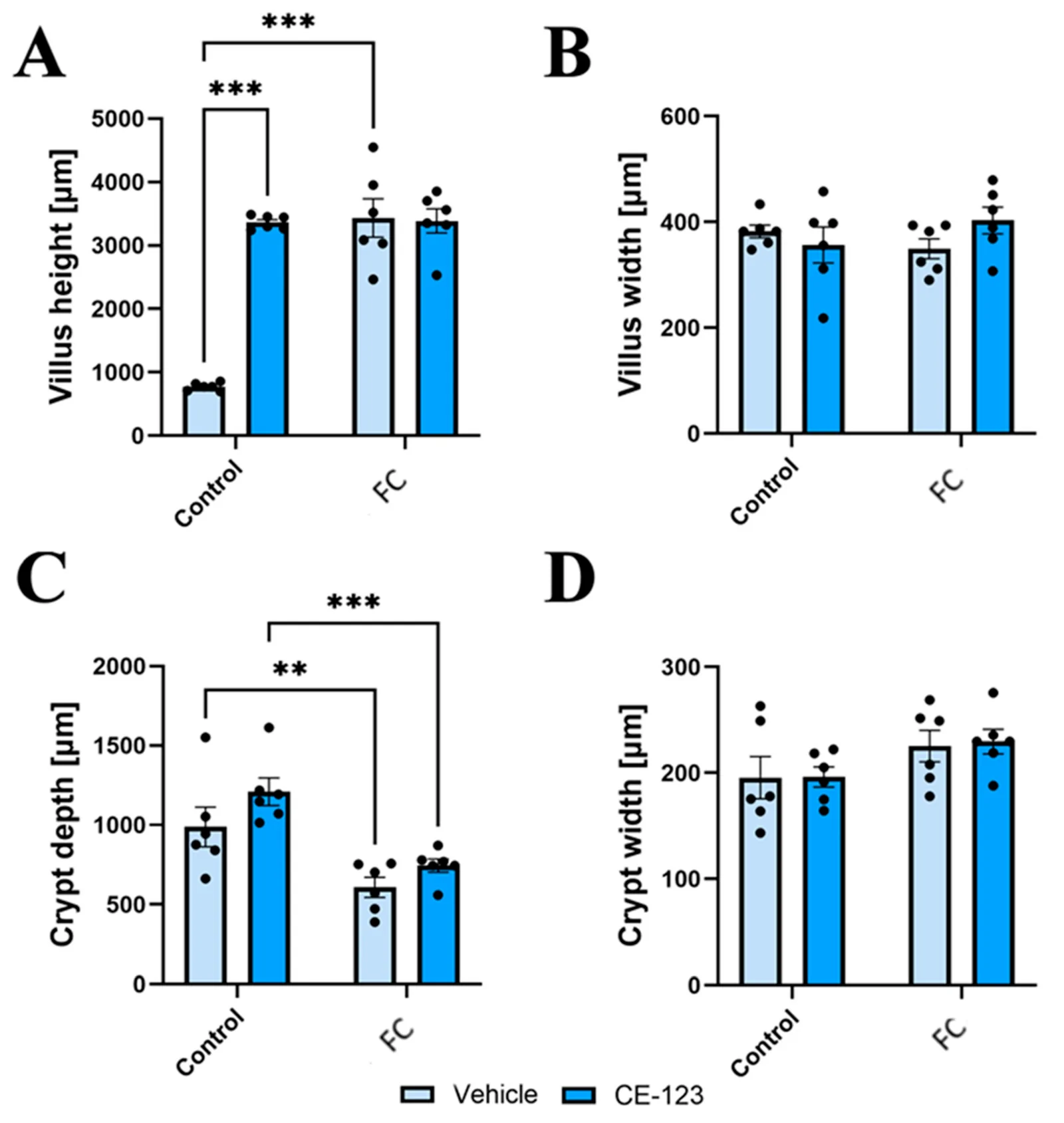
Effects of fear conditioning and CE-123 treatment on morphometric parameters of the duodenal mucosa: (**A**) Villus height, (**B**) villus width, (**C**) crypt depth, and (**D**) crypt width are presented. Animals were assigned to Control or fear-conditioned (FC) groups and received either Vehicle (1% DMSO and 3.3% Tween 80 in 0.9% NaCl) or CE-123. Data are presented as mean ± SEM (*n* = 6 per group). Statistical analysis was performed using two-way ANOVA followed by four prespecified pairwise comparisons for each outcome, with *p*-values adjusted using the Holm–Bonferroni procedure. Asterisks indicate significant differences between groups connected by brackets based on adjusted *p*-values: ** *p* < 0.01; *** *p* < 0.001.

For villus height in the duodenum, a significant FC × CE-123 interaction was observed (F(1,20) = 23.61; *p* < 0.001; Figure 1A). Under control conditions, CE-123 increased villus height compared with Vehicle (*p* < 0.01), while among Vehicle-treated animals, villus height was greater in the FC group than in the Control group (*p* < 0.001). No other pairwise comparisons were significant.

Villus width did not differ significantly among the experimental groups (*p* > 0.05; Figure 1B).

Crypt depth showed significant main effects of FC (F(1,20) = 24.83; *p* < 0.001) and CE-123 treatment (F(1,20) = 4.49; *p* = 0.047; Figure 1C). Crypt depth was lower in the FC + Vehicle group than in the Control + Vehicle group and lower in the FC + CE-123 group than in the Control + CE-123 group (*p* < 0.01 and *p* < 0.001, respectively). No other pairwise comparisons were significant.

Crypt width did not differ significantly among the experimental groups (*p* > 0.05; Figure 1D).

**Figure 2:**
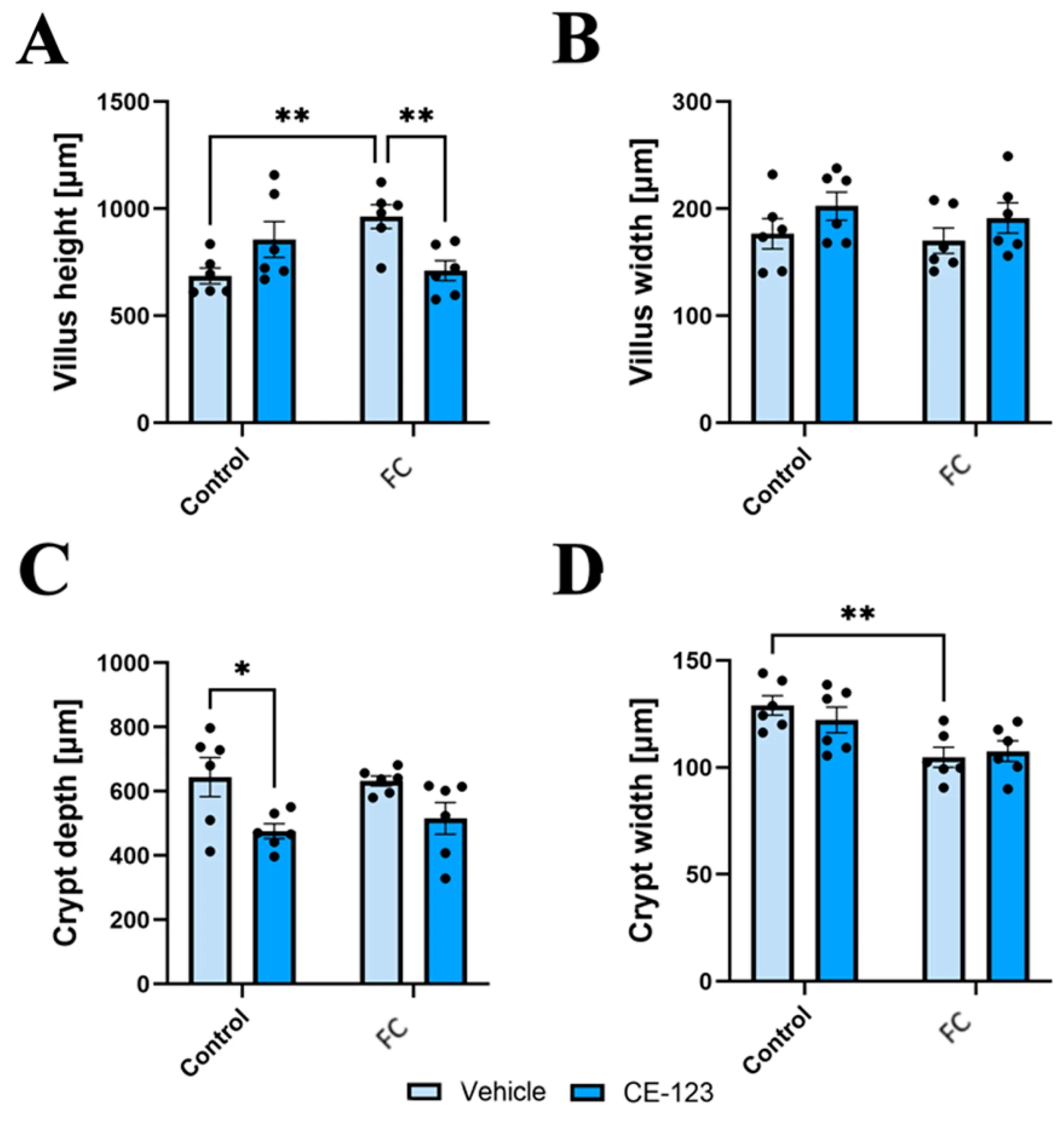
Effects of fear conditioning and CE-123 treatment on morphometric parameters of the jejunal mucosa: (**A**) Villus height, (**B**) villus width, (**C**) crypt depth, and (**D**) crypt width are presented. Animals were assigned to Control or fear-conditioned (FC) groups and received either Vehicle (1% DMSO and 3.3% Tween 80 in 0.9% NaCl) or CE-123. Data are presented as mean ± SEM (*n* = 6 per group). Statistical analysis was performed using two-way ANOVA followed by four prespecified pairwise comparisons for each outcome, with *p*-values adjusted using the Holm–Bonferroni procedure. Asterisks indicate significant differences between groups connected by brackets based on adjusted *p*-values: * *p* < 0.05; ** *p* < 0.01.

For villus height in the jejunum, a significant FC × CE-123 interaction was observed (F(1,20) = 13.03; *p* = 0.002; Figure 2A). Among Vehicle-treated animals, villus height was greater in the FC group than in the Control group (*p* < 0.01), whereas in the FC group, CE-123 treatment was associated with lower villus height than Vehicle (*p* < 0.01). No other pairwise comparisons were significant.

Villus width did not differ significantly among the experimental groups (*p* > 0.05; Figure 2B).

Crypt depth showed a significant main effect of CE-123 treatment (F(1,20) = 11.57; *p* = 0.003; Figure 2C). Crypt depth was lower in the Control + CE-123 group than in the Control + Vehicle group (*p* < 0.05).

For crypt width, a significant main effect of FC was observed (F(1,20) = 14.87; *p* < 0.001; Figure 2D). Crypt width was lower in the FC + Vehicle group than in the Control + Vehicle group (*p* < 0.01).

**Figure 3:**
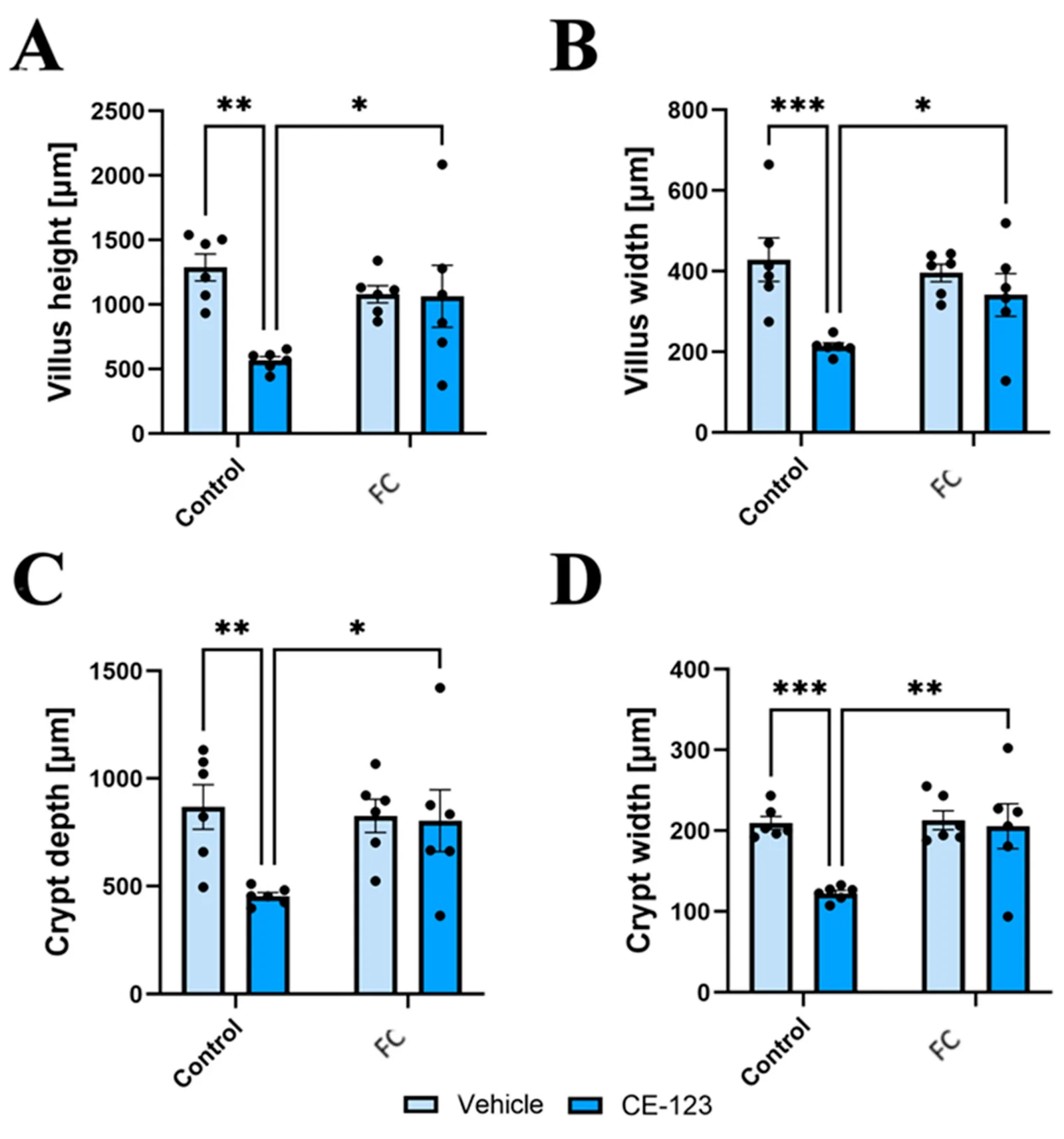
Effects of fear conditioning and CE-123 treatment on morphometric parameters of the ileal mucosa: (**A**) Villus height, (**B**) villus width, (**C**) crypt depth, and (**D**) crypt width are presented. Animals were assigned to Control or fear-conditioned (FC) groups and received either Vehicle (1% DMSO and 3.3% Tween 80 in 0.9% NaCl) or CE-123. Data are presented as mean ± SEM (*n* = 6 per group). Statistical analysis was performed using two-way ANOVA followed by four prespecified pairwise comparisons for each outcome, with *p*-values adjusted using the Holm–Bonferroni procedure. Asterisks indicate significant differences between groups connected by brackets based on adjusted *p*-values: * *p* < 0.05; ** *p* < 0.01; *** *p* < 0.001.

For villus height in the ileum, a significant FC × CE-123 interaction (F(1,20) = 6.73; *p* = 0.017) and a significant main effect of CE-123 treatment (F(1,20) = 7.34; *p* = 0.014; Figure 3A) were observed. Under control conditions, CE-123 decreased villus height compared with Vehicle (*p* < 0.01), whereas among CE-123-treated animals, villus height was greater in the FC group than in the Control group (*p* < 0.05).

Villus width showed a significant main effect of CE-123 treatment (F(1,20) = 11.70; *p* = 0.003; Figure 3B). Villus width was lower in the Control + CE-123 group than in the Control + Vehicle group (*p* < 0.001), whereas among CE-123-treated animals it was greater in the FC group than in the Control group (*p* < 0.05).

Crypt depth also showed a significant main effect of CE-123 treatment (F(1,20) = 5.06; *p* = 0.036; Figure 3C). Crypt depth was lower in the Control + CE-123 group than in the Control + Vehicle group (*p* < 0.01), whereas among CE-123-treated animals it was greater in the FC group than in the Control group (*p* < 0.05).

For crypt width, a significant FC × CE-123 interaction (F(1,20) = 6.42; *p* = 0.020) and significant main effects of FC (F(1,20) = 7.57; *p* = 0.012) and CE-123 treatment (F(1,20) = 9.11; *p* = 0.007; Figure 3D) were observed. Crypt width was lower in the Control + CE-123 group than in the Control + Vehicle group (*p* < 0.001), whereas among CE-123-treated animals it was greater in the FC group than in the Control group (*p* < 0.01). No other pairwise comparisons were significant.

**Figure 4:**
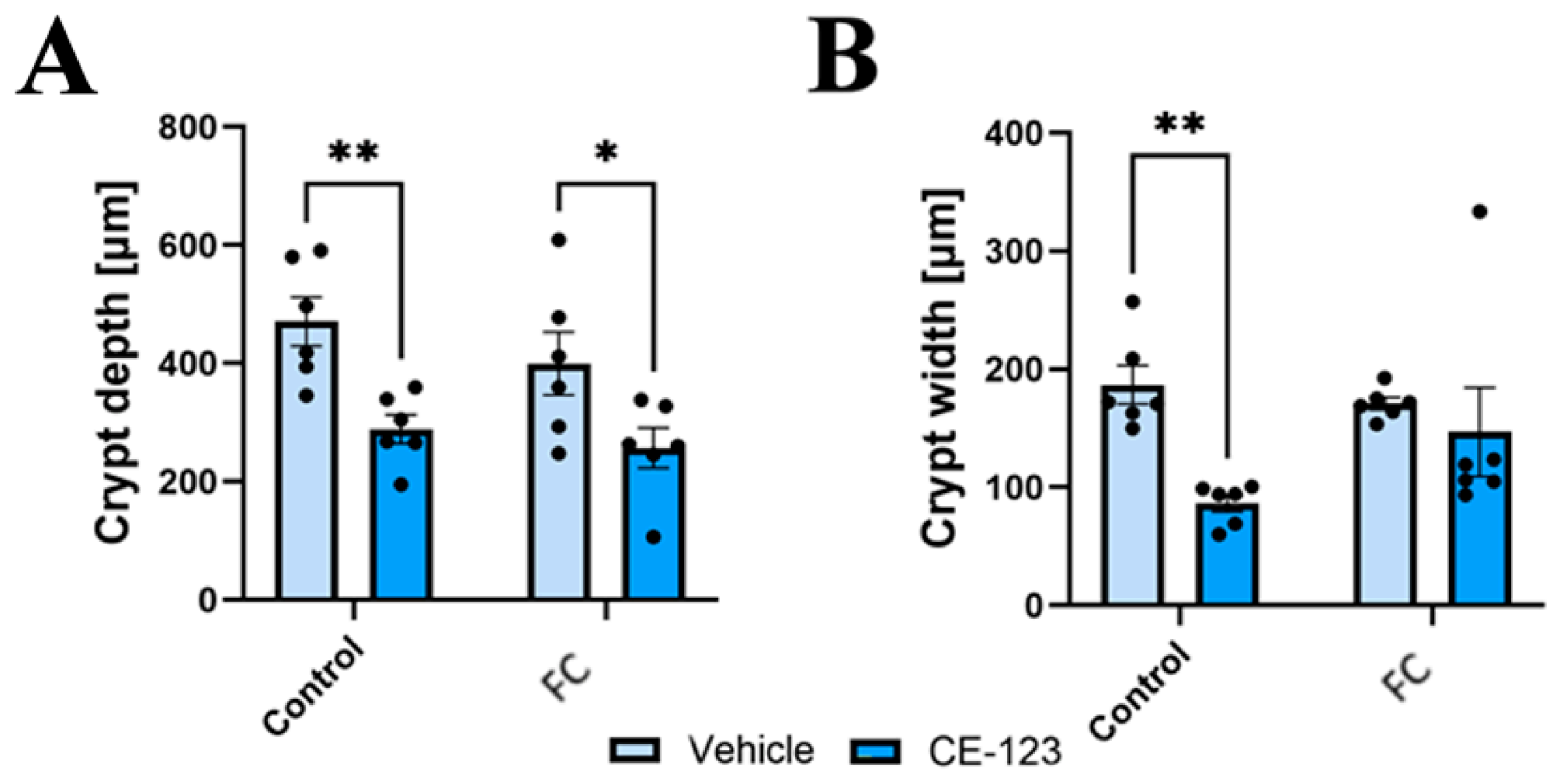
Effects of fear conditioning and CE-123 treatment on morphometric parameters of the colonic mucosa: (**A**) Crypt depth and (**B**) crypt width are presented. Animals were assigned to Control or fear-conditioned (FC) groups and received either Vehicle (1% DMSO and 3.3% Tween 80 in 0.9% NaCl) or CE-123. Data are presented as mean ± SEM (*n* = 6 per group). Statistical analysis was performed using two-way ANOVA followed by four prespecified pairwise comparisons for each outcome, with *p*-values adjusted using the Holm–Bonferroni procedure. Asterisks indicate significant differences between groups connected by brackets based on adjusted *p*-values: * *p* < 0.05; ** *p* < 0.01.

For crypt depth in the colon, a significant main effect of CE-123 treatment was observed (F(1,20) = 16.70; *p* < 0.001; Figure 4A). Crypt depth was lower in the Control + CE-123 group than in the Control + Vehicle group (*p* < 0.01) and lower in the FC + CE-123 group than in the FC + Vehicle group (*p* < 0.05). No other pairwise comparisons were significant.

Crypt width also showed a significant main effect of CE-123 treatment (F(1,20) = 8.89; *p* = 0.007; Figure 4B). Crypt width was lower in the Control + CE-123 group than in the Control + Vehicle group (*p* < 0.01).

### 3.2. Immunoreactivity of CD68, CD206, iNOS, IL-6, IL-10, and MUC2

**Figure 5:**
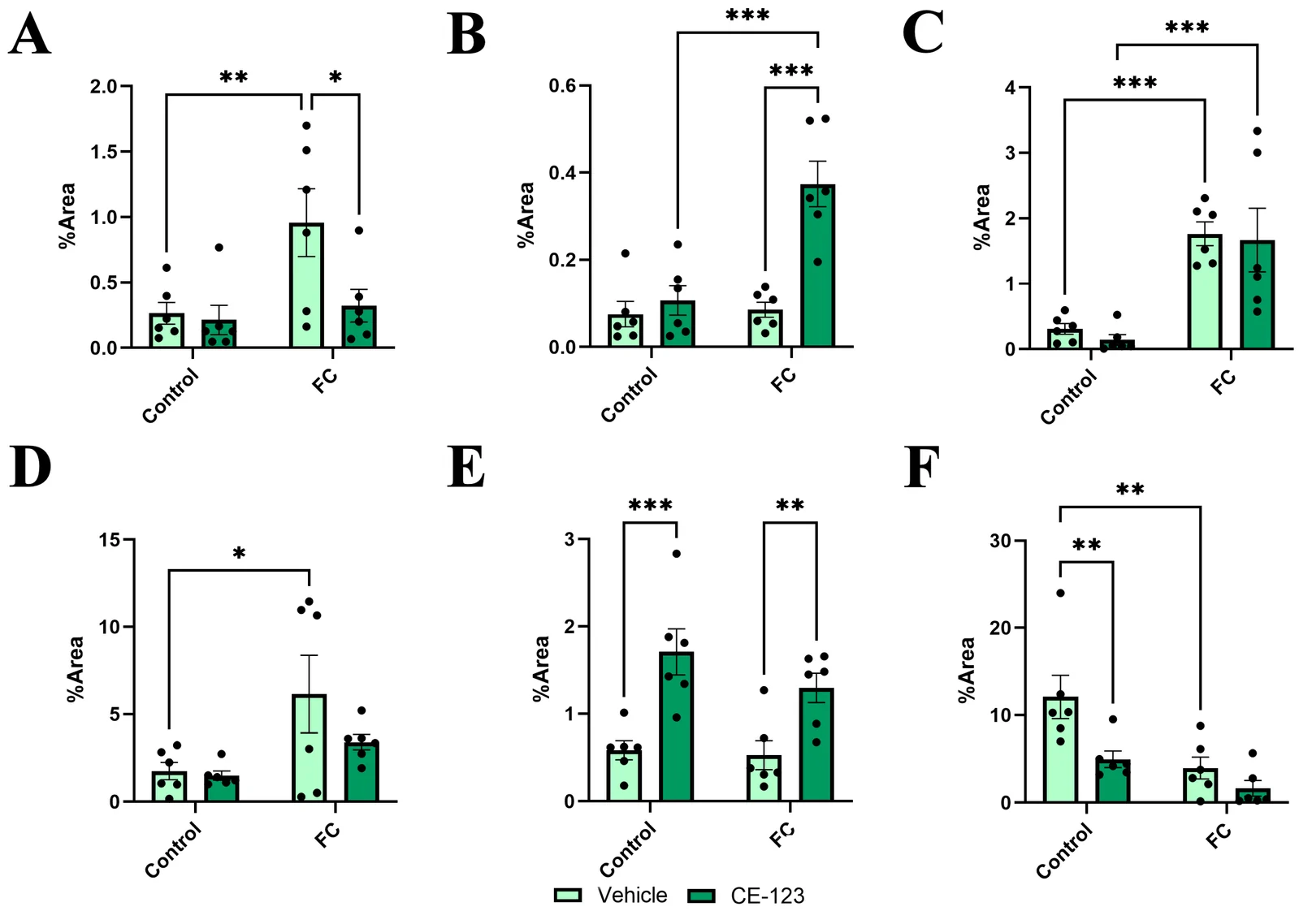
Effects of fear conditioning and CE-123 treatment on the immunoreactivity of selected markers in the duodenal mucosa. The percentage of DAB-positive area (%Area) is shown for (**A**) CD68, (**B**) CD206, (**C**) iNOS, (**D**) IL-6, (**E**) IL-10, and (**F**) MUC2. Animals were assigned to Control or fear-conditioned (FC) groups and received either Vehicle (1% DMSO and 3.3% Tween 80 in 0.9% NaCl) or CE-123. Data are presented as mean ± SEM (*n* = 6 per group). Statistical analysis was performed using two-way ANOVA followed by four prespecified pairwise comparisons for each outcome, with *p*-values adjusted using the Holm–Bonferroni procedure. Asterisks indicate significant differences between groups connected by brackets based on adjusted *p*-values: * *p* < 0.05; ** *p* < 0.01; *** *p* < 0.001.

In the duodenum, CD68 immunoreactivity showed significant main effects of FC (F(1,20) = 6.28; *p* = 0.021) and CE-123 treatment (F(1,20) = 4.60; *p* = 0.044; Figure 5A). CD68 %Area was higher in the FC + Vehicle group than in the Control + Vehicle group (*p* < 0.01) and lower in the FC + CE-123 group than in the FC + Vehicle group (*p* < 0.05).

For CD206, a significant FC × CE-123 interaction was detected (F(1,20) = 13.17; *p* = 0.002), together with significant main effects of FC (F(1,20) = 15.32; *p* < 0.001) and CE-123 treatment (F(1,20) = 20.39; *p* < 0.001; Figure 5B). CD206 %Area was higher in the FC + CE-123 group than in both the FC + Vehicle and Control + CE-123 groups (*p* < 0.001 for both comparisons).

iNOS immunoreactivity was significantly affected by FC (F(1,20) = 31.40; *p* < 0.001; Figure 5C), with higher %Area in both FC groups than in their respective controls (*p* < 0.001 for both comparisons). Similarly, IL-6 showed a significant main effect of FC (F(1,20) = 7.35; *p* = 0.013; Figure 5D), with higher %Area in the FC + Vehicle group than in the Control + Vehicle group (*p* < 0.05).

IL-10 immunoreactivity showed a significant main effect of CE-123 treatment (F(1,20) = 25.92; *p* < 0.001; Figure 5E). IL-10 %Area was higher in the Control + CE-123 group than in the Control + Vehicle group (*p* < 0.001) and in the FC + CE-123 group than in the FC + Vehicle group (*p* < 0.01).

MUC2 immunoreactivity showed significant main effects of FC (F(1,20) = 13.79; *p* = 0.001) and CE-123 treatment (F(1,20) = 9.39; *p* = 0.006; Figure 5F). MUC2 %Area was lower in the Control + CE-123 and FC + Vehicle groups than in the Control + Vehicle group (*p* < 0.01 for both comparisons).

No other pairwise comparisons were significant.

**Figure 6:**
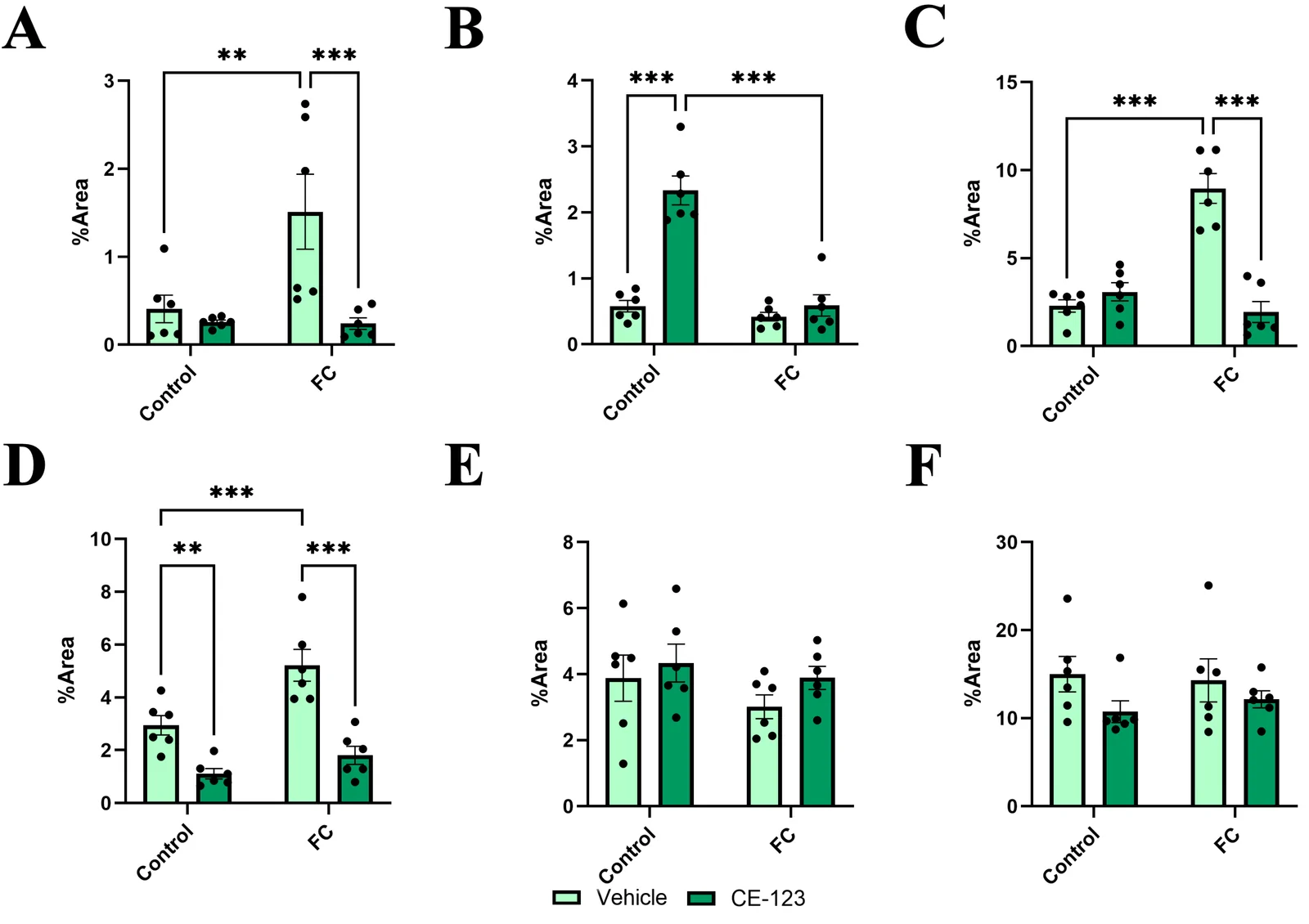
Effects of fear conditioning and CE-123 treatment on the immunoreactivity of selected markers in the jejunal mucosa. The percentage of DAB-positive area (%Area) is shown for (**A**) CD68, (**B**) CD206, (**C**) iNOS, (**D**) IL-6, (**E**) IL-10, and (**F**) MUC2. Animals were assigned to Control or fear-conditioned (FC) groups and received either Vehicle (1% DMSO and 3.3% Tween 80 in 0.9% NaCl) or CE-123. Data are presented as mean ± SEM (*n* = 6 per group). Statistical analysis was performed using two-way ANOVA followed by four prespecified pairwise comparisons for each outcome, with *p*-values adjusted using the Holm–Bonferroni procedure. Asterisks indicate significant differences between groups connected by brackets based on adjusted *p*-values: ** *p* < 0.01; *** *p* < 0.001.

In the jejunum, CD68 immunoreactivity showed a significant FC × CE-123 interaction (F(1,20) = 5.95; *p* = 0.024), together with significant main effects of FC (F(1,20) = 5.63; *p* = 0.028) and CE-123 treatment (F(1,20) = 9.60; *p* = 0.006; Figure 6A). CD68 %Area was higher in the FC + Vehicle group than in the Control + Vehicle group (*p* < 0.01) and lower in the FC + CE-123 group than in the FC + Vehicle group (*p* < 0.001).

For CD206, a significant FC × CE-123 interaction was observed (F(1,20) = 29.25; *p* < 0.001), along with significant main effects of FC (F(1,20) = 42.11; *p* < 0.001) and CE-123 treatment (F(1,20) = 43.11; *p* < 0.001; Figure 6B). CD206 %Area was higher in the Control + CE-123 group than in the Control + Vehicle group and lower in the FC + CE-123 group than in the Control + CE-123 group (*p* < 0.001 for both comparisons).

For iNOS, a significant FC × CE-123 interaction was observed (F(1,20) = 41.96; *p* < 0.001), together with significant main effects of FC (F(1,20) = 20.98; *p* < 0.001) and CE-123 treatment (F(1,20) = 26.48; *p* < 0.001; Figure 6C). iNOS %Area was higher in the FC + Vehicle group than in the Control + Vehicle group and lower in the FC + CE-123 group than in the FC + Vehicle group (*p* < 0.001 for both comparisons).

IL-6 immunoreactivity showed significant main effects of FC (F(1,20) = 13.36; *p* = 0.002) and CE-123 treatment (F(1,20) = 41.88; *p* < 0.001; Figure 6D). IL-6 %Area was lower in the Control + CE-123 group than in the Control + Vehicle group (*p* < 0.01), higher in the FC + Vehicle group than in the Control + Vehicle group (*p* < 0.001), and lower in the FC + CE-123 group than in the FC + Vehicle group (*p* < 0.001).

No significant differences among the experimental groups were observed for IL-10 or MUC2 immunoreactivity (Figure 6E,F).

**Figure 7:**
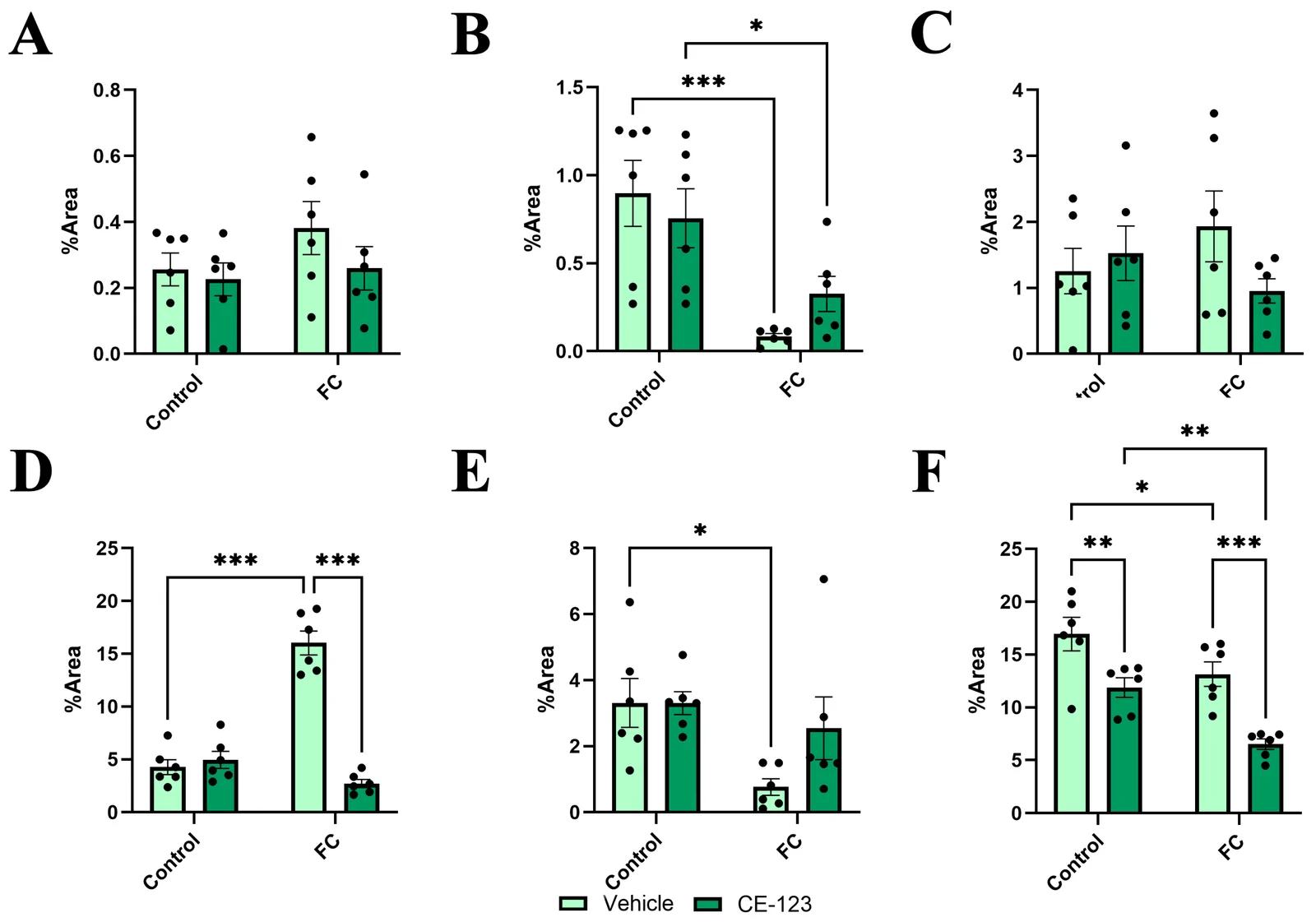
Effects of fear conditioning and CE-123 treatment on the immunoreactivity of selected markers in the ileal mucosa. The percentage of DAB-positive area (%Area) is shown for (**A**) CD68, (**B**) CD206, (**C**) iNOS, (**D**) IL-6, (**E**) IL-10, and (**F**) MUC2. Animals were assigned to Control or fear-conditioned (FC) groups and received either Vehicle (1% DMSO and 3.3% Tween 80 in 0.9% NaCl) or CE-123. Data are presented as mean ± SEM (*n* = 6 per group). Statistical analysis was performed using two-way ANOVA followed by four prespecified pairwise comparisons for each outcome, with *p*-values adjusted using the Holm–Bonferroni procedure. Asterisks indicate significant differences between groups connected by brackets based on adjusted *p*-values: * *p* < 0.05; ** *p* < 0.01; *** *p* < 0.001.

In the ileum, CD68 immunoreactivity did not differ significantly among the experimental groups (Figure 7A).

CD206 immunoreactivity showed a significant main effect of FC (F(1,20) = 21.01; *p* < 0.001; Figure 7B). CD206 %Area was lower in the FC + Vehicle group than in the Control + Vehicle group (*p* < 0.001) and lower in the FC + CE-123 group than in the Control + CE-123 group (*p* < 0.05). No other pairwise comparisons were significant.

No significant differences among the groups were observed for iNOS immunoreactivity (Figure 7C).

For IL-6, a significant FC × CE-123 interaction was detected (F(1,20) = 75.71; *p* < 0.001), together with significant main effects of FC (F(1,20) = 34.98; *p* < 0.001) and CE-123 treatment (F(1,20) = 61.61; *p* < 0.001; Figure 7D). IL-6 %Area was higher in the FC + Vehicle group than in the Control + Vehicle group (*p* < 0.001) and lower in the FC + CE-123 group than in the FC + Vehicle group (*p* < 0.001).

IL-10 immunoreactivity showed a significant main effect of FC (F(1,20) = 6.73; *p* = 0.017; Figure 7E), with lower %Area in the FC + Vehicle group than in the Control + Vehicle group (*p* < 0.05).

MUC2 immunoreactivity showed significant main effects of FC (F(1,20) = 16.87; *p* < 0.001) and CE-123 treatment (F(1,20) = 27.46; *p* < 0.001; Figure 7F). MUC2 %Area was lower in the Control + CE-123 group than in the Control + Vehicle group (*p* < 0.01), lower in the FC + Vehicle group than in the Control + Vehicle group (*p* < 0.05), lower in the FC + CE-123 group than in the Control + CE-123 group (*p* < 0.01), and lower in the FC + CE-123 group than in the FC + Vehicle group (*p* < 0.001).

**Figure 8:**
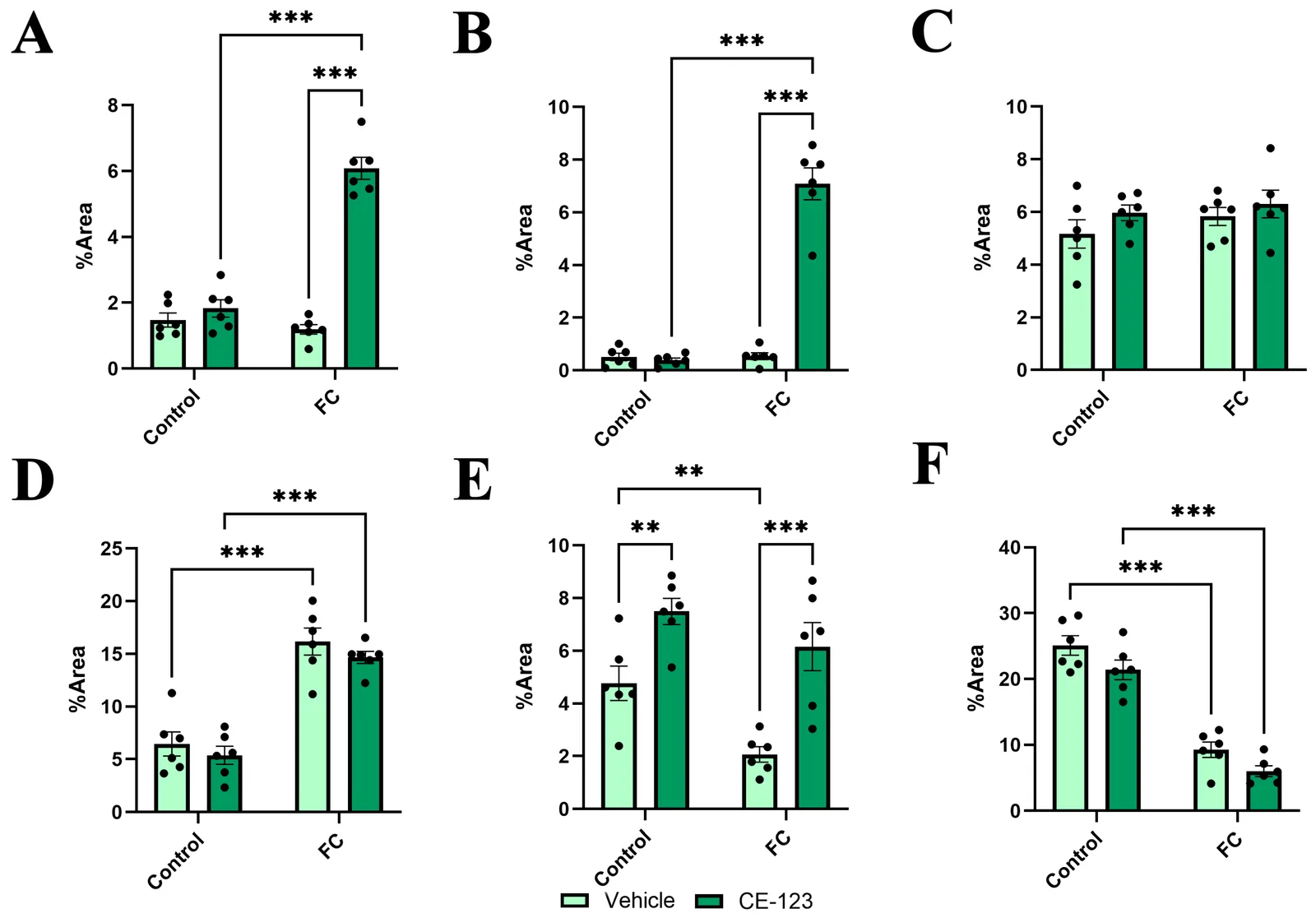
Effects of fear conditioning and CE-123 treatment on the immunoreactivity of selected markers in the colonic mucosa. The percentage of DAB-positive area (%Area) is shown for (**A**) CD68, (**B**) CD206, (**C**) iNOS, (**D**) IL-6, (**E**) IL-10, and (**F**) MUC2. Animals were assigned to Control or fear-conditioned (FC) groups and received either Vehicle (1% DMSO and 3.3% Tween 80 in 0.9% NaCl) or CE-123. Data are presented as mean ± SEM (*n* = 6 per group). Statistical analysis was performed using two-way ANOVA followed by four prespecified pairwise comparisons for each outcome, with *p*-values adjusted using the Holm–Bonferroni procedure. Asterisks indicate significant differences between groups connected by brackets based on adjusted *p*-values: ** *p* < 0.01; *** *p* < 0.001.

In the colon, CD68 immunoreactivity showed a significant FC × CE-123 interaction (F(1,20) = 83.86; *p* < 0.001), together with significant main effects of FC (F(1,20) = 64.33; *p* < 0.001) and CE-123 treatment (F(1,20) = 111.8; *p* < 0.001; Figure 8A). CD68 %Area was higher in the FC + CE-123 group than in both the FC + Vehicle and Control + CE-123 groups (*p* < 0.001 for both comparisons).

A similar pattern was observed for CD206, with a significant FC × CE-123 interaction (F(1,20) = 108.7; *p* < 0.001) and significant main effects of FC (F(1,20) = 110.3; *p* < 0.001) and CE-123 treatment (F(1,20) = 100.7; *p* < 0.001; Figure 8B). CD206 %Area was higher in the FC + CE-123 group than in both the FC + Vehicle and Control + CE-123 groups (*p* < 0.001 for both comparisons).

No significant differences among the experimental groups were observed for iNOS immunoreactivity (Figure 8C).

IL-6 immunoreactivity showed a significant main effect of FC (F(1,20) = 90.56; *p* < 0.001; Figure 8D). IL-6 %Area was higher in the FC + Vehicle group than in the Control + Vehicle group and in the FC + CE-123 group than in the Control + CE-123 group (*p* < 0.001 for both comparisons).

For IL-10, significant main effects of FC (F(1,20) = 10.24; *p* = 0.004) and CE-123 treatment (F(1,20) = 29.16; *p* < 0.001; Figure 8E) were observed. IL-10 %Area was higher in the Control + CE-123 group than in the Control + Vehicle group (*p* < 0.01), lower in the FC + Vehicle group than in the Control + Vehicle group (*p* < 0.01), and higher in the FC + CE-123 group than in the FC + Vehicle group (*p* < 0.001).

MUC2 immunoreactivity showed significant main effects of FC (F(1,20) = 150.8; *p* < 0.001) and CE-123 treatment (F(1,20) = 7.41; *p* = 0.013; Figure 8F). MUC2 %Area was lower in the FC + Vehicle group than in the Control + Vehicle group and in the FC + CE-123 group than in the Control + CE-123 group (*p* < 0.001 for both comparisons; Table 2).

## 4. Discussion

The present study showed that fear conditioning followed by extinction was associated with marked but strongly region-dependent alterations in the intestinal mucosa, involving morphometric parameters, selected immune markers, and MUC2 immunoreactivity. The response to CE-123 was likewise dependent on the intestinal segment and prior fear-conditioning history. Importantly, morphometric, immunological, and MUC2-related changes did not always occur in parallel, indicating that different components of mucosal homeostasis may respond differently to aversive conditioning and subsequent extinction. Such regional heterogeneity is biologically plausible because individual intestinal segments differ in their immune organization, microbiota and luminal environment, and enteric innervation [30,31,32,33]. Previous studies similarly indicate that intestinal responses to stress depend on both the nature of the stressor and the gastrointestinal region examined [34,35].

The morphometric changes observed following fear conditioning and extinction were not unidirectional. In FC + Vehicle animals, villus height was increased in the duodenum and jejunum, accompanied by reduced crypt depth in the duodenum and reduced crypt width in the jejunum. These findings suggest remodeling of the crypt–villus architecture rather than simple mucosal atrophy. Although villus height is commonly regarded as an indicator related to potential absorptive surface, it does not directly reflect absorptive capacity [36]. Previous stress studies have also produced divergent morphometric findings. Increased duodenal villus height has been reported in young rats subjected to chronic restraint stress [37], whereas other stress models have shown reduced villus height and increased crypt depth [12,38]. Such differences may reflect variation in the type and duration of stress, intestinal region, and timing of tissue collection. Changes in crypt and villus morphology may also develop asynchronously during stress exposure [11].

In contrast, prior fear conditioning was associated with relatively limited morphometric changes in the ileum, whereas CE-123 affected several parameters under control conditions. CE-123 reduced villus height and width as well as crypt dimensions in the control ileum and reduced crypt depth in the colon under both Control and FC conditions. These findings indicate that CE-123 did not simply reverse conditioning-associated alterations but also influenced intestinal morphology independently of prior fear-conditioning history. The underlying mechanism remains unclear. Dopaminergic signaling participates in the regulation of gastrointestinal motility, secretion, epithelial transport, mucosal protection, and immune function [22,39], making indirect effects of DAT inhibition on mucosal architecture plausible, although this was not directly investigated here. Regional differences were particularly evident in the immunohistochemical findings. In the duodenum and jejunum, FC + Vehicle animals showed increased immunoreactivity of CD68, iNOS, and IL-6, consistent with enhancement of a local response with a pro-inflammatory component. Psychological stress can influence intestinal immunity through neuroendocrine pathways and enteric neuroimmune interactions [13,40]. In particular, chronic psychological stress has been shown to modify the intestinal immune environment and promote recruitment of monocyte-derived cells [13]. The ileum showed a different profile. Prior fear conditioning was associated with markedly increased IL-6 immunoreactivity together with reduced CD206 and IL-10, whereas CD68 and iNOS remained unchanged. A similar dissociation was evident in the colon, where increased IL-6 and reduced IL-10 occurred without changes in CD68 or iNOS. These results indicate that increased immunoreactivity of an inflammatory mediator does not necessarily coincide with parallel changes in all inflammation-associated markers. Moreover, IL-6 can originate from several mucosal cell populations and therefore should not be interpreted as a direct indicator of macrophage activation [41]. Likewise, changes in CD206 and iNOS should not be equated with a simple shift between classical M1 and M2 macrophage phenotypes, particularly because intestinal macrophages comprise heterogeneous populations strongly shaped by their local environment [42,43]. The observed changes are therefore more appropriately interpreted as alterations in the local immune profile.

Among the examined regions, the jejunum showed the clearest CE-123-associated attenuation of the pro-inflammatory component. CE-123 reduced the conditioning-associated increases in CD68, iNOS, and IL-6 immunoreactivity. In the duodenum, CE-123 reduced CD68 and increased CD206 and IL-10, but did not significantly alter iNOS or IL-6 within the FC groups. Thus, even in the proximal small intestine, its effects were selective rather than uniformly anti-inflammatory.

Dopaminergic regulation of immune cells may contribute to these findings. Functional DAT expression has been reported in monocytes and macrophages, including a subset of intestinal macrophages [24], while dopamine can either enhance or suppress cytokine production depending on receptor subtype, concentration, and cellular activation state [25]. This indicates that the effects of dopaminergic modulation on immune responses cannot be simply classified as pro- or anti-inflammatory. Interestingly, pharmacological DAT inhibition has been reported to increase IL-6 and TNF-α secretion in LPS-stimulated macrophages [24], whereas CE-123 reduced several inflammation-associated markers in the jejunum in the present study. This discrepancy suggests that the intestinal effects observed here cannot be explained simply by direct DAT inhibition in macrophages. CE-123 crosses the blood–brain barrier and modifies central dopaminergic signaling [44,45], raising the possibility that some intestinal effects are mediated indirectly through brain–gut communication. The present study, however, cannot distinguish central from peripheral mechanisms.

MUC2 immunoreactivity further emphasized the regional nature of the response. It remained unchanged in the jejunum despite increased CD68, iNOS, and IL-6, whereas it was reduced in FC animals in the duodenum, ileum, and colon. In the ileum, CE-123 reduced IL-6 but further decreased MUC2, indicating that changes in these two parameters were not directly coupled. MUC2 production and secretion are regulated by goblet-cell function, immune signals, and the microbiota [17,46], and IL-10 may support proper MUC2 folding and secretion by limiting endoplasmic reticulum stress [47]. Accordingly, alterations in MUC2 may occur partly independently of changes in individual inflammatory mediators.

In the present study, CE-123 did not significantly increase IL-10 immunoreactivity in the ileum despite the marked reduction in IL-6, further suggesting that modulation of the inflammatory response and alterations in the mucus layer may occur partly independently. This distinct response may also be related to the unique immunological organization of the ileum, including well-developed gut-associated lymphoid tissue and region-specific immune cell populations [30]. This may partly explain why prior fear conditioning was associated with marked changes in IL-6, CD206, and IL-10 immunoreactivity in the ileum despite relatively limited morphometric alterations.

The regional differences in MUC2 may be particularly relevant in the colon, where MUC2 constitutes the major structural component of the mucus layer separating the epithelium from the dense luminal microbiota [48,49]. The effects of stress on MUC2 have also been demonstrated previously in animal models. Shigeshiro et al. (2012) showed that repeated water-immersion stress in rats reduced colonic *Muc2* expression through both a decrease in goblet cell number and reduced *Muc2* expression at the single-cell level [18], consistent with the reduced colonic MUC2 immunoreactivity observed in the present study. However, MUC2 abundance alone does not reflect the functional properties of the mucus barrier. Da Silva et al. (2014) showed that water-avoidance stress altered mucin O-glycosylation, mucus properties, and intestinal permeability despite unchanged goblet cell number and *Muc2* expression [50]. Accordingly, the reduced MUC2 immunoreactivity—mucosal component of the intestinal barrier here shouldn’t be interpreted as direct evidence of impaired intestinal permeability.

The most complex response to CE-123 was observed in the colon. In FC animals, CE-123 increased CD68, CD206, and IL-10 immunoreactivity, but did not reduce IL-6 or restore MUC2 immunoreactivity, while iNOS remained unchanged. Thus, CE-123 did not simply suppress the inflammatory response but rather modified selected components of the local immune profile. The increases in CD68 and CD206 should nevertheless be interpreted cautiously, because intestinal macrophages represent heterogeneous functional populations that cannot be unequivocally classified according to the classical M1/M2 framework [42]. Moreover, the present immunohistochemical approach does not establish whether changes in CD68 and CD206 occurred within the same cells. The coexistence of increased IL-10 with persistently elevated IL-6 further supports this interpretation. Pro-inflammatory and regulatory responses are not mutually exclusive, and IL-10 may limit excessive inflammatory activation while pro-inflammatory signaling persists [21]. CE-123 may therefore have enhanced the regulatory component of the colonic immune response without fully suppressing conditioning-associated alterations. Such context-dependent effects are consistent with the complex immunomodulatory actions of dopamine [25].

The effects of CE-123 were also evident in control animals. The compound increased IL-10 immunoreactivity in the duodenum and colon and CD206 in the jejunum, while reducing MUC2 immunoreactivity in the duodenum and ileum. These findings indicate that CE-123 may directly modulate selected mechanisms of the immune response and the intestinal mucosal component, with the nature of these effects varying between intestinal regions. Thus, CE-123 did not act solely by reversing conditioning-associated changes, but rather exerted effects that depended on both the intestinal region and prior fear-conditioning history. Such regional differences may, at least in part, reflect the functional specialization of the intestinal immune environment [20,40]. The timing of CE-123 administration is also relevant. Treatment began during the fear-extinction phase, after initial conditioning and sensitization, and continued through the subsequent stages of the paradigm. Its effects should therefore be interpreted as modulation of responses following prior aversive exposure rather than as a preventive action. Because dopamine contributes to fear-extinction learning [23] and CE-123 was administered before extinction-related behavioral sessions, the intestinal effects may reflect both peripheral actions of DAT inhibition and indirect consequences of altered central responses during fear extinction and subsequent aversive procedures.

Taken together, the intestinal response following fear conditioning and extinction did not follow a simple sequence linking aversive experience, inflammation, and mucus-barrier alterations. In the jejunum, increased CD68, iNOS, and IL-6 occurred without changes in MUC2; in the ileum, increased IL-6 and reduced CD206 and IL-10 coincided with decreased MUC2 despite unchanged CD68 and iNOS; and in the colon, increased IL-6 and reduced MUC2 occurred without increased iNOS. Morphometric changes also did not consistently parallel immunological or MUC2-related alterations. These findings further support a region-dependent and partly dissociated mucosal response. Likewise, CE-123 cannot be regarded as uniformly anti-inflammatory. Its clearest attenuation of inflammation-associated markers occurred in the jejunum, whereas responses in the duodenum, ileum, and colon were more complex. Overall, the findings support a context-dependent immunomodulatory action of CE-123 that varies according to intestinal segment and prior fear-conditioning history [25].

Several limitations should be considered. First, the experimental paradigm was designed to investigate responses associated with fear conditioning and extinction rather than to reproduce the full clinical phenotype of PTSD. Moreover, the Control and FC groups differed primarily in exposure to the high-intensity cue-paired foot shocks during the initial conditioning session, whereas subsequent lower-intensity aversive procedures were applied to all animals. Accordingly, between-group differences should be interpreted in relation to prior fear-conditioning history rather than as the general effects of stress exposure. Quantitative immunohistochemical analysis based on the percentage of DAB-positive area does not identify the cellular sources of the observed signals. This is particularly relevant for CD68, CD206, iNOS, IL-6, and IL-10 and precludes direct conclusions regarding macrophage abundance or polarization. Future studies using multiplex immunofluorescence or other cell-specific approaches could provide more precise characterization of the local immune response. MUC2 immunoreactivity also represents only one component of the intestinal mucus barrier and does not provide a direct measure of permeability or functional barrier integrity. Barrier function additionally depends on mucus organization, mucin secretion and glycosylation, tight junctions, and other epithelial mechanisms. Finally, only adolescent female rats were examined. Sex, sex hormones, and developmental stage may influence HPA-axis reactivity, intestinal barrier function, and responses to stress [51,52]. It has been demonstrated that estrogens may modulate the stress response through activation of the HPA axis, which may be related to the close anatomical proximity of CRF receptors (CRFR) and estrogen receptors (ER) within the hypothalamus. Moreover, CRF expression has been shown to increase following activation of both ERα and ERβ. It should also be noted that, following exposure to stressors, rats develop visceral hypersensitivity, which was completely abolished following OVX [53]. Furthermore, an increased frequency of functional gastrointestinal disorders (FGIDs) has been reported in women, particularly during the follicular phase, which is characterized by relatively high estrogen concentrations [54]. It is also well established that estradiol modulates tight junction (TJ) function in the endothelium and reproductive tissues, while estrogen receptors are expressed in the intestinal epithelium. Braniste et al. demonstrated that estrogens strengthen the intestinal epithelial barrier by regulating occludin and junctional adhesion molecule-A (JAM-A) expression [55]. The present findings therefore cannot be directly generalized to males or animals at other developmental stages. Another limitation of the present study is the lack of individual food-intake measurements. Although body weight was monitored throughout the experimental period, the absence of corresponding food-intake data precludes a definitive assessment of whether nutritional or growth-related factors contributed to the observed alterations in intestinal villus and crypt morphology.

## 5. Conclusions

In summary, fear conditioning followed by extinction was associated with region-specific alterations in intestinal mucosal morphology, local immune markers, and MUC2 immunoreactivity. Changes in morphometry and inflammation-associated markers were most prominent in the duodenum and jejunum, whereas alterations in regulatory markers and reduced MUC2 were more evident in the ileum and colon. CE-123 showed its clearest attenuation of the pro-inflammatory component in the jejunum, while its effects in other regions were more complex. Overall, selective DAT inhibition with CE-123 exerted a context-dependent immunomodulatory rather than uniformly anti-inflammatory effect, and the morphometric, immune, and MUC2-related components of the intestinal response did not necessarily change in parallel.

## Figures and Tables

**Table 1:** Primary and secondary antibodies used in the study.

Target Antigen	Antibody Type	Host Species	Manufacturer (Country)	Catalog Number	Dilution
Anti-mouse/rabbit	Polyclonal, HRP- conjugated, secondary	Goat	ImmunoLogic Arnhem, Netherlands	DPVB-HRP	RTU ^1^
CD68	Polyclonal, primary	Rabbit	Proteintech, Rosemont, IL, USA	28058-1-AP	1:500
CD206	Polyclonal, primary	Rabbit	Proteintech, Rosemont, IL, USA	18704-1-AP	1:3000
iNOS	Polyclonal, primary	Rabbit	Proteintech, Rosemont, IL, USA	22226-1-AP	1:500
IL-6	Polyclonal, primary	Rabbit	Novus Biologicals, Minneapolis, MN, USA	NB600-1131	1:400
IL-10	Polyclonal, primary	Rabbit	Affinity Biosciences, Cincinnati, OH, USA	DF6894	1:50
MUC2	Polyclonal, primary	Rabbit	Affinity Biosciences, Cincinnati, OH, USA	DF8390	1:200

^1^ Ready to use.

**Table 2:** Summary of segment-specific changes in intestinal mucosal morphometry and immunoreactivity following fear conditioning and CE-123 treatment.

Group	Parameter	Duodeum	Jejunum	Ileum	Colon
FC + Vehicle vs. Control + Vehicle	Villus height [µm]	↑	↑	→	x
	Villus width [µm]	→	→	→	x
	Crypts depth [µm]	↓	→	→	→
	Crypts width [µm]	→	↓	→	→
	CD68 [%Area]	↑	↑	→	→
	CD206 [%Area]	→	→	↓	→
	iNOS [%Area]	↑	↑	→	→
	IL-6 [%Area]	↑	↑	↑	↑
	IL-10 [%Area]	→	→	↓	↓
	MUC2 [%Area]	↓	→	↓	↓
FC + CE-123 vs. Control + CE-123	Villus height [µm]	→	→	↑	x
	Villus width [µm]	→	→	↑	x
	Crypts depth [µm]	↓	→	↑	→
	Crypts width [µm]	→	→	↑	→
	CD68 [%Area]	→	→	→	↑
	CD206 [%Area]	↑	↓	→	↑
	iNOS [%Area]	→	→	→	→
	IL-6 [%Area]	→	→	→	↑
	IL-10 [%Area]	→	→	→	→
	MUC2 [%Area]	→	→	↓	↓
FC + Ce-123 vs. FC + Vehicle	Villus height [µm]	→	↓	→	x
	Villus width [µm]	→	→	→	x
	Crypts depth [µm]	→	→	→	↓
	Crypts width [µm]	→	→	→	→
	CD68 [%Area]	↓	↓	→	↑
	CD206 [%Area]	↑	→	→	↑
	iNOS [%Area]	→	↓	→	→
	IL-6 [%Area]	→	↓	↓	→
	IL-10 [%Area]	↑	→	→	↑
	MUC2 [%Area]	→	→	↓	→
Control + CE-123 vs. Control + Vehicle	Villus height [µm]	→	→	↑	x
	Villus width [µm]	→	→	↑	x
	Crypts depth [µm]	→	→	↑	→
	Crypts width [µm]	→	→	↑	→
	CD68 [%Area]	→	→	→	→
	CD206 [%Area]	→	↑	→	→
	iNOS [%Area]	→	→	→	→
	IL-6 [%Area]	→	↓	→	→
	IL-10 [%Area]	↑	→	→	↑
	MUC2 [%Area]	↓	→	↓	→

The schematic summarizes the direction of changes in morphometric parameters (villus height, villus depth, crypt depth, and crypt width; µm) and immunoreactivity parameters expressed as the percentage of DAB-positive area (%Area) for CD68, CD206, iNOS, IL-6, IL-10, and MUC2 across the duodenum, jejunum, ileum, and colon. Animals were assigned to Control or fear-conditioned (FC) groups and received either Vehicle (1% DMSO and 3.3% Tween 80 in 0.9% NaCl) or CE-123. Data are presented as mean ± SEM (*n* = 6 per group). Statistical analysis was performed using two-way ANOVA followed by four prespecified pairwise comparisons for each outcome, with *p*-values adjusted using the Holm–Bonferroni procedure. Asterisks indicate significant differences between groups connected by brackets based on adjusted *p*-values: *p* < 0.05; *p* < 0.01 for morphometric parameters, and *p* < 0.01; *p* < 0.001 for immunoreactivity parameters. Arrow legend: ↑—significant increase; ↓—significant decrease; → no significant change.

## Data Availability

The raw data supporting the conclusions of this article will be made available by the authors upon request.

## Abbreviations

The following abbreviations are used in this manuscript:

PTSD	post-traumatic stress disorder
HPA	hypothalamic–pituitary–adrenal
DAT	dopamine transporter
FC	fear conditioning
DAB	3,3′-diaminobenzidine
iNOS	inducible nitric oxide synthase
IL-6	interleukin-6
IL-10	interleukin-10
MUC2	mucin 2
HRP	horseradish peroxidase
ROI	region of interest
